# Management of potato brown rot disease using chemically synthesized CuO-NPs and MgO-NPs

**DOI:** 10.1186/s40529-023-00393-w

**Published:** 2023-07-17

**Authors:** Amira Rabea, E. Naeem, Naglaa M. Balabel, Ghadir E. Daigham

**Affiliations:** 1 Bacterial Disease Research Department, Plant Pathology Research Institute, Agricultural Research Center (ARC), Giza, Egypt; 2 Department of Botany and Microbiology, Faculty of Science, Al-Azhar University (Girls Branch), Cairo, Egypt; 3 Potato Brown Rot Project, Ministry of Agriculture, Dokki, Giza, Egypt

**Keywords:** Brown rot disease, Chlorophyll, CuO-NPs, Lipid peroxidation, MgO-NPs, Potato, *R. solanacearum*

## Abstract

**Background:**

Potatoes are a crucial vegetable crop in Egypt in terms of production and consumption. However, the potato industry suffers significant annual losses due to brown rot disease. This study aimed to suppress *Ralstonia solanacearum* (*R. solanacearum*), the causative agent of brown rot disease in potatoes, using efficient and economical medications such as CuO and MgO metal oxide nanoparticles, both in vitro and in vivo, to reduce the risk of pesticide residues.

**Results:**

CuO and MgO metal oxide nanoparticles were synthesized via a simple chemical process. The average particle size, morphology, and structure of the nanoparticles were characterized using UV-visible spectroscopy, transmission electron microscopy (TEM), zeta potential analysis, X-ray diffraction (XRD), and Fourier transform infrared (FTIR) spectroscopy. The growth of *R. solanacearum* was strongly inhibited by CuO and MgO NPs at a concentration of 3 mg/mL, resulting in zones of inhibition (ZOI) of 19.3 mm and 17 mm, respectively. The minimum inhibitory concentration (MIC) and minimum bactericidal concentration (MBC) of CuO-NPs and MgO-NPs were 0.5, 0.6, and 0.6, 0.75 mg/mL, respectively. When applied in vivo through seed dressing and tuber soaking at their respective MIC concentrations, CuO-NPs and MgO-NPs significantly reduced the incidence of brown rot disease to 71.2% and 69.4%, respectively, compared to 43.0% and 39.5% in bulk CuSO_4_ and bulk MgSO_4_ treatments, respectively. Furthermore, CuO-NPs and MgO-NPs significantly increased the yield, total chlorophyll content, and enzyme efficiency of potato plants compared with the infected control plants. TEM revealed that the bacterial cytomembrane was severely damaged by nanomechanical forces after interaction with CuO-NPs and MgO-NPs, as evidenced by lipid peroxidation and ultrastructural investigations.

**Conclusion:**

The results of this study suggest that CuO-NPs and MgO-NPs can be used as intelligent agents to manage plant pathogens in agriculture. The use of metal oxide nanoparticles could provide a risk-free alternative for treating plant diseases, which are currently one of the biggest challenges faced by the potato industry in Egypt. The significant increase in yield, photosynthetic pigments, enzymatic activity, and total phenol-promoted resistance to *R. solanacearum* in potato plants treated with CuO-NPs and MgO-NPs compared to infected control plants highlights the potential benefits for the potato industry in Egypt. Further investigations are needed to explore using metal oxide nanoparticles for treating other plant diseases.

## Background

Potato (*Solanum tuberosum*), a crucial vegetable crop in Egypt, is one of the most important crops worldwide. It ranks fourth among the most significant staple foods consumed globally, following wheat, rice, and maize. Egypt produces over 6.4 million tons of potatoes annually from 209 thousand hectares, with an average yield of approximately 30.6 tons per hectare and a market value of approximately $307 million (FAOSTAT 2020). According to Mahgoub et al. (2015), Egypt is the top African producer and exporter of potatoes. Vegetable cultivation accounts for approximately 20% of all agricultural land in 25 of Egypt’s 27 governorates (Rabia et al. 2021).

Globally, potato plantations are threatened by a severe disease known as brown rot, which causes an annual loss of more than $950 million (Elazouni et al. 2019). The bacterial pathogen responsible for this disease, *R. solanacearum*, survives in various hosts and affects fifty-four plant families, totaling over 450 plant species (Patil et al. 2017). No resistant crop varieties are currently available (Ahmed et al. 2022). Moreover, increased pesticide use can adversely affect non-target species (Chhipa 2019). Therefore, it is essential to find alternative techniques, such as metal oxide nanoparticles and other nanotechnologies, to minimize the harmful effects of pesticides on the environment. Copper (Cu) is one of the seven essential micronutrients, whereas magnesium (Mg) is one of the three secondary macronutrients required for plant development and growth. Mg plays a crucial role in photosynthesis as the central atom in the chlorophyll molecule and is involved in most enzyme activation processes. Cu participates in numerous enzymatic reactions, metabolic processes, and oxidation-reduction reactions in many biological systems of plant. Cu and Mg are essential mineral components in several physiological processes related to plant virulence, pathogenesis, and defense.

The benefits of magnesium include increased tissue ability to fight disease, which can reduce disease spread and improve plant absorption of nutrients from the soil, including nitrogen (N), phosphorus (P), and potassium (K) (Huber and Jones 2013). Among the various inorganic metal oxides, copper oxide and magnesium oxide nanoparticles (CuO-NPs and MgO-NPs) have several advantages: they are nontoxic, less expensive, relatively simple to obtain and prepare, require minimal doses, exhibit thermal stability, and are not cytotoxic or genotoxic to humans. Moreover, the United States Food and Drug Administration has recently recognized them as safe materials. There is evidence that CuO-NPs have antibacterial activity against various bacterial species.

CuO-NPs have demonstrated antibacterial activity against several bacterial species, including *Staphylococcus aureus*, *Bacillus subtilis*, *Pseudomonas aeruginosa*, and *Escherichia coli* (Azam et al. 2012). Compared with certain antibiotics, copper nanoparticles have shown high efficacy against common and clinical gram-positive bacterial strains, such as methicillin-resistant *Staphylococcus aureus* (Kruk et al. 2015). MgO nanoparticles (MgO-NPs) have been shown to distort and damage *E. coli* cell membranes, release intracellular materials, and cause subsequent cell death (Jin and He 2011). In culture media, MgO-NPs demonstrated strong antibacterial activity against *S. aureus* (Bindhu et al. 2016).

CuO-NPs and MgO-NPs have recently demonstrated enormous potential in agriculture as excellent choices for plant protection (Imada et al. 2016; Elmer et al. 2018). For example, CuO-NPs prevent the growth of Phytophthora, an oomycete (Giannousi et al. 2013). Vanathi et al. (2016) showed that CuO-NPs with a diameter of 28.4 nm exhibited antifungal efficacy against several phytopathogens in the following decreasing order: *Fusarium culmorum*, *Aspergillus niger*, *F. oxysporum*, *A. flavus*, and *A. fumigatus*. Additionally, chemically produced CuO-NPs have been shown to be effective antifungals against *Alternaria alternata*, *Phoma destructive*, *Curvularia lunata* and *F. oxysporum* (Kanhed et al. 2014), as well as *Omphalia* sp on coffee tree (Agredo-Trochez et al. 2022).

Previous studies have shown that MgO-NPs are effective against various plant pathogenic fungi, including *F. oxysporum* (Abdel-Aziz et al. 2020). In a separate investigation, Sierra-Fernandez et al. (2017) explored the antifungal properties of MgO nanoparticles to preserve the calcareous stone heritage. However, there is limited knowledge regarding the antibacterial capabilities of CuO and MgO nanoparticles against plant pathogenic bacteria (Cai et al. 2018).

Nanoparticles can be produced through various processes, including chemical, physical, and biological approaches, with chemical methods being the most widely used commercially (Phiwdang et al. 2013). The co-precipitation technique is a crucial chemical process used to synthesize nano metal oxides, enabling the production of large quantities of nanoparticles in a short time with high control over size and distribution (Manyasree et al. 2018). By adjusting the concentration of chemicals and regulating the reaction conditions, nanoparticles of different shapes can be formed through chemical processes (Tomonari et al. 2008).

## Materials and methods

All chemicals, including copper nitrate, polyvinylpyrrolidone (PVP), sodium hydroxide, and magnesium sulfate, were purchased from Sigma-Aldrich (St. Louis, MO, USA). Louis, MO, USA). *R. solanacearum* was previously isolated and molecularly identified by Elazouni et al. (2019).

### An initial test to investigate the effectiveness of bulks on *R. solanacearum*

Seven different concentrations (0.05, 0.2, 0.4, 0.6, 1.0, 3.0, and 5.0 mg/mL) of CuSO_4_ and MgSO_4_ were prepared and used in this study. To prepare antibacterial stocks, 10 mg of each tested agent was dissolved in a suitable solvent. A suspension of each concentration was applied to *R. solanacearum*. The plates were divided into four replicates and treated with gentamicin or sterile water as a control. All plates were maintained at a temperature of 28 ± 2 °C and relative humidity of 80 ± 5%. After two days, the bacterial count was recorded using the method described by Tiwari et al. (2017).

### Synthesis of copper oxide nanoparticles

In addition, with some modifications, 2.9 g of copper nitrate and 1.2 g of polyvinylpyrrolidone (PVP) were dissolved in 100 mL of deionized water using a magnetic stirrer for approximately two hours. The solution was then heated to 60 °C. Then, 90 mL of 1 M sodium hydroxide (NaOH) was added dropwise to the aqueous copper nitrate solution with vigorous stirring for an hour and a half until a black-brown precipitate formed. The precipitate was centrifuged at 10,000 rpm for 30 min using a Rotina 380 R centrifuge (Hettich, Tuttlingen, Germany) and placed in an oven at 50 °C for 2 h (Baqer et al. 2018).

### Synthesis of magnesium oxide nanoparticles

The co-precipitation method was employed using the Taguchi approach to synthesize magnesium oxide nanoparticles. First, 1 M aqueous solutions of sodium hydroxide (NaOH) and magnesium sulfate were prepared in 100 mL of distilled water. The two solutions were then added dropwise to the solution at a rate of 1 mL/min while stirring at high speed for 1 h. The resulting solid solution was then thoroughly cleaned using deionized water to remove any impurities and dried in an oven at approximately 100 °C for over 24 h. Finally, the product was annealed for 3 h at 450 °C to yield white MgO nanoparticles (Imani and Safaei 2019).

### Techniques for material characterization of NPs

*UV-vis spectroscopy* The NP products were analyzed by UV-vis spectroscopy at the Nanotechnology and Advanced Materials Central Lab (NAMCL) of the Agricultural Research Center in Egypt. UV-vis scanning was performed using a Jasco V630 instrument produced in Europe, and the analysis range was set between 200 and 800 nm.

### Transmission electron microscopy (TEM)

The morphology, size, and form of the nanoparticles were analyzed using TEM at the National Research Center in Giza, Egypt. TEM analysis was conducted using two instruments: a Philips CM 12 TEM operated at 120 KV with resolutions up to 510,000 magnifications and a Joel 1230 TEM operated at 100 KV coupled to a CD camera (JEOL, Tokyo, Japan). SAED and HRTEM imaging were performed using a JEOL 3000 F instrument operated at 300 KV with a spot size of 1 nm and an acquisition time of one minute, respectively.

### Zetasizer (ZS)

The Nano ZS from Malvern, UK, was utilized in all analyses, which were performed at the Central Laboratory for Nanotechnology and Advanced Materials (NAMCL), Agricultural Research Center (ARC), Giza, Egypt. The laser particle analyzer (LPA) was carried out using the Malvern Dispersion Software, with a range of zeta potential (mV) from − 200 to 200 mV and a size range (nm) from 0.6 to 6000, as reported by Li et al. (2017).

### X-ray diffraction (XRD)

Powder elemental analysis was conducted using a Rigaku Smart Lab Beijing Co. XRD model (Beijing, China). The measurements were performed with a wavelength of 1.54 Å, 45 kV, and 40 mA under a temperature range between 4 and 80° in the angular range of 2θ. The interplanar spacing (‘d’) was calculated using Bragg’s law, 2d sinθ =  λ, where ‘θ’ is the diffraction angle and ‘λ’ is the wavelength of the X-ray used.

### Fourier transform infrared spectroscopy (FTIR)

In addition, FTIR analysis was performed using a Nicolet Avatar 660 FTIR analyzer (Nicolet, USA) at the National Petroleum Research Institute in Egypt, covering a range of 500–4000 cm^-1^.

### Bactericidal action of nanoparticles on *R. solanacearum*

This study employed the agar diffusion technique to examine the antimicrobial activity of the nanoparticles. The antimicrobial activity was indicated by the zone of inhibition, as defined by Bonev et al. (2008). Cell suspensions of *R. solanacearum* were prepared using 24-h-old bacterial cells containing 10^–7^ colony-forming units per mL (CFU/mL). Sterilized YPG agar medium was thoroughly mixed with 100 µL of the cell suspension and evenly distributed across sterilized Petri dishes. Aqueous nanoparticles (3 mg/mL) with well diameters of 6.0 mm were tested in 100 µL and added to the medium after solidification. The plates were incubated at 30 °C for 48 h, followed by a 2-h pre-incubation at 4 °C. The inhibition zones were measured in millimeters after incubation. Each test was repeated five times for each nanoparticle, along with a control group (gentamicin). Acute toxicity was evaluated after two days to identify inhibition. Before treatment, all the solutions were sonicated for 5 min to reduce sedimentation, particularly at higher doses.

### Measurement of the minimum inhibitory and bactericidal concentrations of NPs against *R. solanacearum*

The measurements were performed by repeated half-fold dilutions of NPs from a diluted stock solution in Mueller-Hinton broth medium, following the method outlined by Azam et al. (2012), with slight modifications. NPs were initially prepared as solutions in PVP at 1 mg/mL to ensure complete solubilization. Eleven sterile tubes were prepared, each containing 5 mL Mueller-Hinton broth. The first tube received 3 mg/mL of the NPs solution, while the remaining tubes received various dilutions of the NPs solution to obtain concentrations ranging from 3 to 0.27 mg/mL (3, 1.5, 1.0, 0.75, 0.6, 0.5, 0.43, 0.38, 0.33, 0.3, and 0.27 mg/mL). A 0.5 McFarland turbidity standard bacterial suspension was created using fresh cultures of the standards and serially diluted. Each tube was supplemented with positive and negative controls. The tubes were then incubated at 30 °C for 24 h. The absence of turbidity after incubation, indicating the inhibition of bacterial growth, was used to identify the antibacterial activities of the NPs in the tubes. The minimum inhibitory concentration (MIC) was defined as the highest dilution with no turbidity. Subsequently, 100 µL of each MIC was inoculated onto plates and incubated at 30 °C for 24 h to calculate the bacterial count in CFU/mL using the plate count method (Tiwari et al. 2017) in the presence of NPs. The diameter of the inhibition zone was measured in millimeters for each MIC (Bonev et al. 2008). To calculate the minimum bactericidal concentration (MBC), 100 µL of each MIC concentration and concentrations below and above the MIC values were added to nutrient agar. The plates were incubated at 30 °C after an initial two-hour incubation at 4 °C (Ruparelia et al. 2008).

### Lipid analysis for investigating the impact of NPs on *R. solanacearum*

The procedure outlined by Roy Choudhury et al. (2012) was followed to extract total lipids, with modifications. Briefly, dried bacterial material (0.5 g) (treated with NPs, along with control sets) was homogenized for 2 min using a Waring blender homogenizer (Type RQ-127 A) with 10 mL of methanol, chloroform, and water mixture (2:1:0.8 v/v). To eliminate any residual water, the lower chloroform phase was separated and concentrated at 30–35 °C by adding benzene and using a rotating vacuum evaporator. The resulting residue was dissolved in the required volume of chloroform. Phospholipids were estimated as a percentage of the total lipids using Kates’ method (Kates 1972).

### Investigating the impact of NPs on *R. solanacearum *using TEM preparations

TEM was performed at the Central Laboratory of the Electron Microscope, Fungi Center, El-Azhar University, Egypt.

### Influence of compost and humic acid on the persistence of *R. solanacearum *in the presence of NPs

The compost product used in this study, produced by the Ministry of Agriculture in Giza, Egypt, contained compound percentages of organic matter (38.6%), organic carbon (22.4%), C/N ratio (10.5%), total phosphorus (0.26%), and potassium (1.1%). These compounds exert beneficial effects on plant growth. Before application, the compost tea preparation was examined for microorganisms on the PDA medium. Compost tea and humic acid (15.9%) were added at 250 mL/pot before planting. After the soil was infested with the pathogen, pots were sown with disease-free tubers of the Spunta variety. Each treatment was replicated five times, and uninoculated controls were prepared similarly.

Bacterial counting was performed to assess the effect of product mixtures on the longevity of *R. solanacearum* in the soil. The density of *R. solanacearum* was recorded for each treatment. The soil samples were transferred to the laboratory for bacterial counts in potato pots using the SMSA medium (Elphinstone et al. 1996).

### Effect of NPs on potato brown rot disease incidence and severity in vivo

The experiment was conducted during the winter season of 2020, using a completely randomized block design in pots under greenhouse conditions with five repetitions. The pots were filled with a mixture of sandy and clay soil (1:1, v/v) collected from potato districts in El-Sharkia Governorate, Egypt. Each pot contained 6 kg of soil, and one uniform disease-free tuber of the Spunta variety was planted in each pot. The soil infestation was performed using the soil drenching method of Deberdt et al. (2012) at a rate of 180 mL/pot of *R. solanacearum*, corresponding to a rate of 30 mL/kg of soil. The pathogen density was adjusted to approximately 10^7^ cells/mL. The study followed the steps suggested by Tahir et al. (2017), with some modifications.

### Preparation of *R. solanacearum* inoculums

*R. solanacearum* isolates were propagated in liquid YPG broth and incubated for four days at 28 °C on a rotary shaker (New Brunswick Innova 44, Eppendorf, USA) at 150 rpm. Subsequently, the cultures were centrifuged at 10 °C for 10 min at 10,000 rpm using a centrifuge (Rotina 380 R, Hettich, Tuttlingen, Germany). The resulting bacterial pellets were optically calibrated to 10^8^ CFU/mL and suspended in distilled water. Measurements were taken using a spectrophotometer (BioSpec-mini, Shimadzu, Kyoto, Japan) at an optical density (OD) of 600.

### Experimental steps

First, a group of potato tubers was soaked in a nanoparticle solution for 30 min before planting. After 21 days of planting, the pots were infected with *R. solanacearum*. One day after infection, nanoparticles were added again to the infected pots at 180 mL/pot, equivalent to 30 mL/kg soil (Chen et al. 2019). Another group of potato tubers was treated with 5 g of commercial biocide (Rhizo-N powder, BIOTEC Company for fertilizers and biocides, Egypt). The main ingredient of the biocide was *Bacillus subtilis* (30 × 10^6^ CFU/mL). The application rate was 1 g/kg of seed.

Uninfected controls (healthy plants) and controls infested with *R. solanacearum* were included in the experiment. Throughout the growing season, all pots were placed in a greenhouse with a temperature range of 26–30 °C and a relative humidity of 75–90%. After 85 days of potato planting, various parameters were observed, including the reduction percentage, wilt incidence, and yield per plant. Data on disease severity as a percentage, infection rate, tuber weight (in grams), phenol levels, enzyme activity (polyphenol oxidase and peroxidase), and pigments (chlorophyll a, b, and carotenoids) were collected.

### Determination of the pigments involved in photosynthetic processes

Photosynthetic pigments (chlorophyll a, chlorophyll b, and carotenoids) were extracted from fresh leaf samples in the abovementioned experiments. The extraction was performed using pure acetone based on Fadeel’s method (Fadeel 1962). Spectrophotometric analysis was used to estimate the optical density of the filtrate at specific wavelengths. The wavelengths used were 662 nm for chlorophyll a, 644 nm for chlorophyll b, and 440.5 nm for carotenoids.

### Analyzing the activity of peroxidase (PO)

Peroxidase (EC 1.11.1.7) activity was determined and expressed in milligrams per gram of fresh weight (mg/g fwt). The activity was measured by recording the change in absorbance per minute immediately after adding the substrate, following the method described by Kochba et al. (1977). Color density was measured using a spectrophotometer (Milton Roy Spectronic 601, Rochester, New York, USA) at a wavelength of 425 nm. Readings were taken every 30 s for 10 readings.

### Analyzing the activity of polyphenol oxidase (PPO)

Polyphenol oxidase (EC 1.14.18.1) activity was measured using the method outlined by Lisker et al. (1983). Color density was measured using a spectrophotometer (Milton Roy Spectronic 601, Rochester, New York, USA) at a wavelength of 495 nm. Ten readings were taken every 30 s, for 10 readings.

### Extraction and determination of total phenols

The color optical density of the reaction mixture was measured at a wavelength of 520 nm using a spectrophotometer (Milton Roy Spectronic 601, Rochester, New York, USA), following the method described by Mechikova et al. (2007).

### Statistical analysis

The obtained data were analyzed using ANOVA, and the least significant difference (LSD) test was used to determine statistically significant differences between treatments at a significance level of p < 0.05. Statistical analysis was performed using the CoStat software (CoHort, Monterey, CA, USA). The results were presented as mean ± standard error with a sample size (n) of 3, following the guidelines described by Snedecor and Cochran (1980).

## Results

### An initial test to investigate the effectiveness of bulks on *R. solanacearum*

This experiment aimed to investigate the effects of two metals, CuSO_4_ and MgSO_4_, on the growth of *R. solanacearum*. As shown in **(**Table 1**)**, bacterial growth decreased with increasing metal concentrations. In contrast, low metal concentrations resulted in the narrowest growth density compared to that of the control.

**Table 1:** Effect of CuSO_4_ and MgSO_4_ salts as bulk on the growth of virulent *R. solanacearum*

Treatment	Concentration (mg/mL)						
	0.05	0.2	0.4	0.6	1	3	5
	Log CFU/mL						
CuSO _4_	6.8c ± 0.346	6.2b ± 0.404	4.7b ± 0.346	4.2b ± 0.346	3.6b ± 0.289	0.00 ± 0.00	0.00b ± 0.00
MgSO _4_	8.6b ± 0.289	6.9b ± 0.462	4.8b ± 0.289	4.8b ± 0.346	3.9b ± 0.346	0.00b ± 0.00	0.00b ± 0.00
Control	9.8a ± 0.289	9.8a ± 0.289	9.8a ± 0.289	9.8a ± 0.289	9.8a ± 0.289	9.8a ± 0.289	9.8a ± 0.289
LSD at 5%	1.07	1.35	1.06	1.14	1.07	n.s	N.S

Means ± SE, (a, b, …), data with the different letter are significant, while the data with the same letter within the same column are not significantly different (p < 0.05)

### Characterization of CuO-NPs

#### UV-visible spectroscopy

**Fig. 1:**
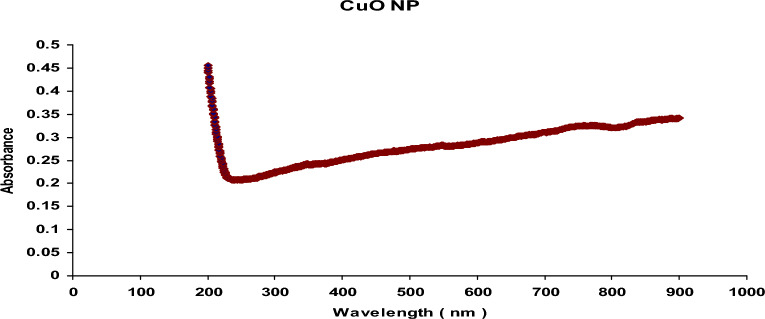
UV-vis spectrum of the synthesized CuO-NPs, showing a peak at approximately 220 nm with an absorption value of 0.2

Ultraviolet (UV) spectroscopy revealed the reduction of CuO to CuO nanoparticles, which was confirmed by the peak obtained at 220 nm with an absorption value of 0.2 (Fig. 1).

#### Transmission electron microscopy (TEM)

**Fig. 2:**
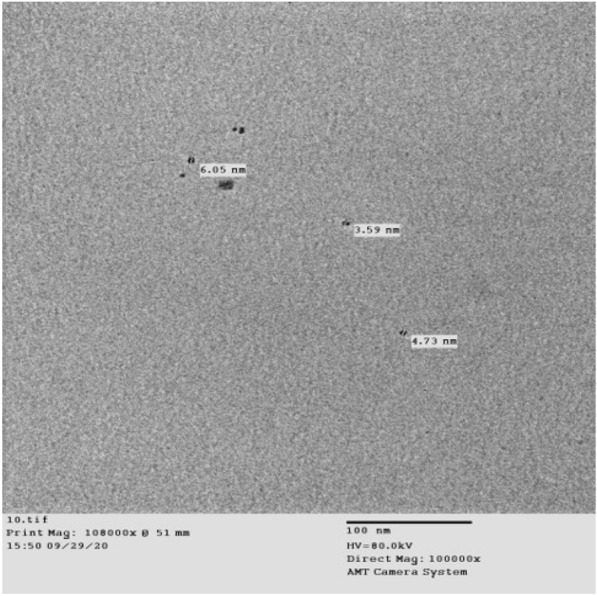
TEM photograph of CuO-NPs showing smooth, spherical, and loosely distributed NPs with particle sizes ranging from 3.59 to 6.05 nm, homogeneously distributed

The synthesized CuO-NPs had an average size of 3.59 and 6.05 nm. They were smooth, spherical, loose, and evenly distributed throughout the matrix (Fig. 2).

### Zeta potential

**Fig. 3:**
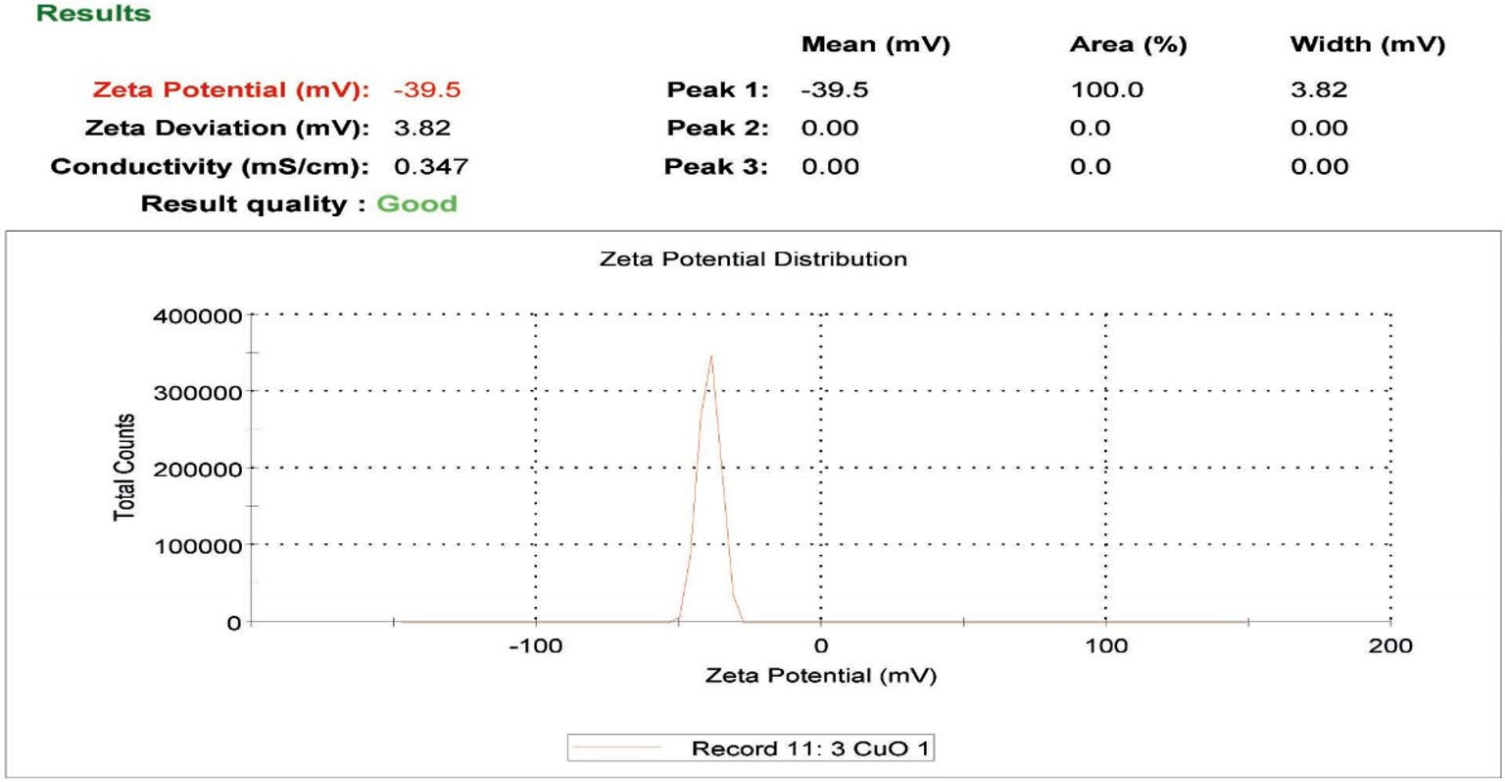
Histogram of the zeta potential distribution of CuO-NPs, showing a zeta potential of -39 mV

The zeta potential of the CuO-NPs was measured to be − 39.5 mV, indicating the surface charge of the particles. This value suggested that CuO-NPs were stable, as shown in (Fig. 3).

### FTIR analysis

**Fig. 4:**
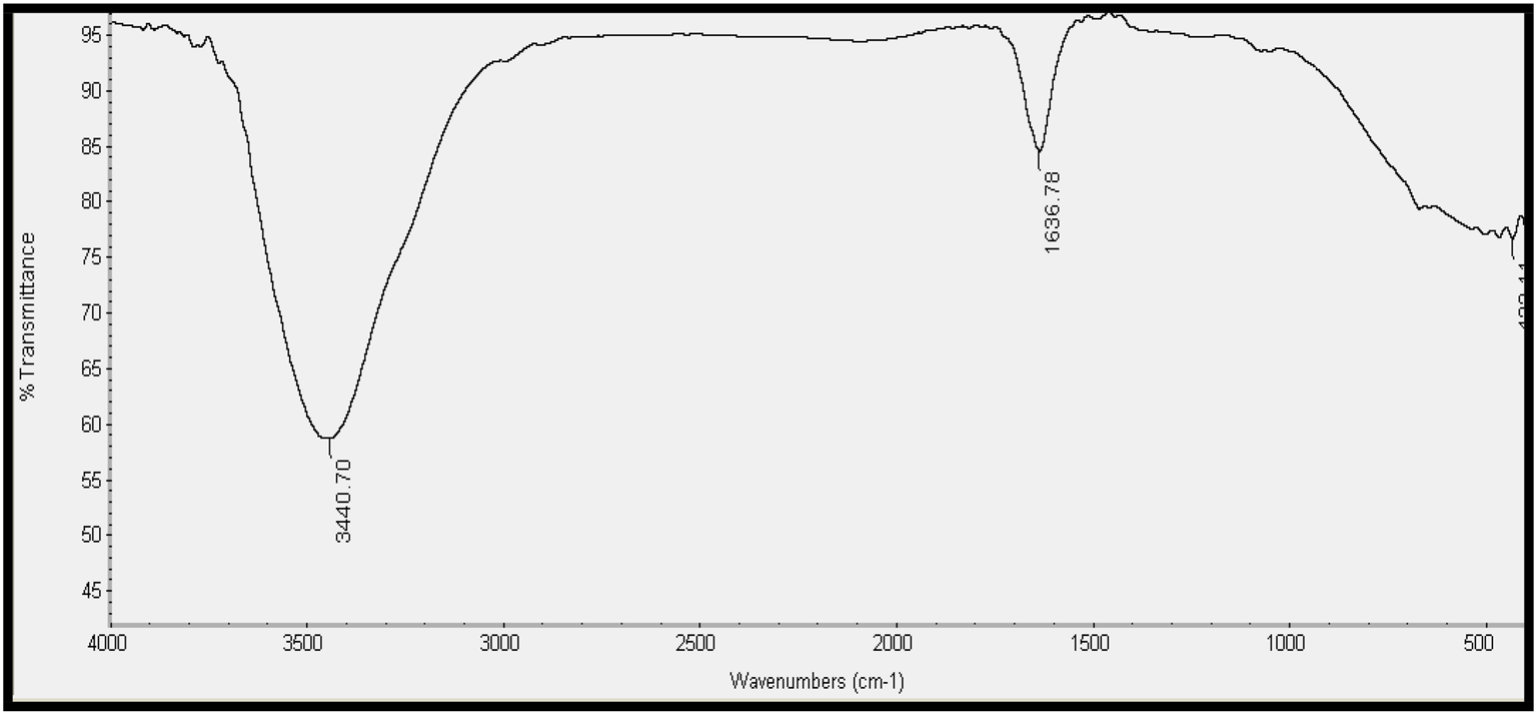
FTIR spectra of CuO-NPs measured at wavenumbers of 400–4000 cm^− 1^. Strong bands were visible at 3440.7, 1636.78, and 432.11 cm^− 1^

Strong bands were observed in the CuO-NP spectra at wavelengths of 3440.7, 1636.78, and 432.11 cm^− 1^ (Fig. 4). The band at 3440.7 cm^− 1^ is attributed to the O–H stretching of phenols and alcohols with hydrogen bonds. The presence of amides I and II, as well as NO_2_ stretching of the nitro compound, was indicated by the band at 1636.78 cm^− 1^. These vibrations arise from the vibrational carbonyl stretch and –N–H stretch of proteins’ amide linkages. The peak at 432.11 cm^− 1^ in the fingerprint region represents a mixture of various vibrations, making it challenging to pinpoint a specific compound. The FTIR spectra of the copper nanoparticles suggested the presence of several organic compounds in their vicinity.

### XRD

**Fig. 5:**
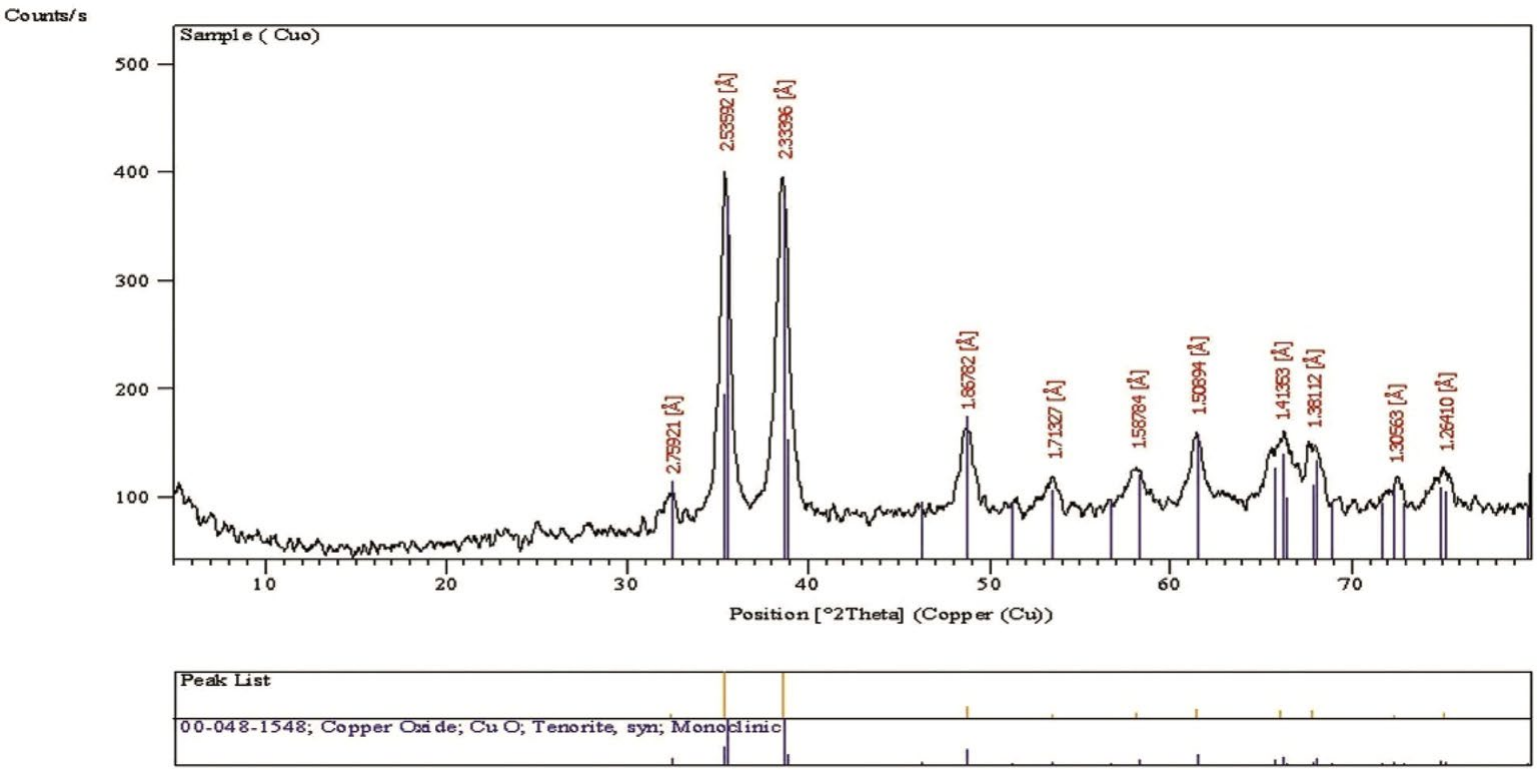
XRD spectra of CuO nanopowder used to detect its crystalline nature. It shows two sharp peaks at 2θ of 35.544° and 38.709° with reference code (00-048-1548)

The X-ray diffraction patterns of the synthesized CuO-NPs were analyzed to determine their crystalline nature. Distinct diffraction peaks were observed at specific 2θ values. Reference code 00-048-1548 was used to identify the crystal structure. The diffraction peaks observed at 35.417°, 35.544°, 38.709°, 38.902°, 48.718°, and 61.526° correspond to the crystal planes (h k l) of (0 0 2), (1 1 –1), (1 1 1), (2 0 0), and (2 0 –2) crystal planes, respectively. Two sharp peaks were particularly prominent at 35.544° and 38.709°, as depicted in (Fig. 5**)**.

### Characterization of MgO-NPs

#### UV-visible spectroscopy

**Fig. 6:**
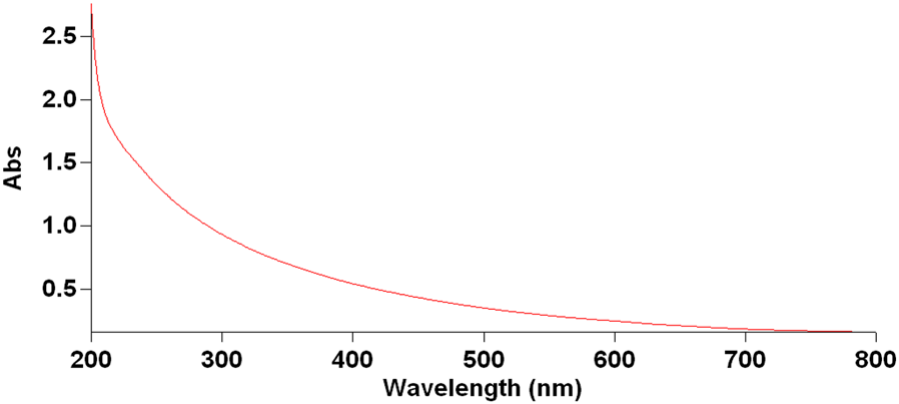
UV-vis absorption spectra of synthesized MgO-NPs showing maximum surface plasmon resonance (SPR) at 200 nm

Ultraviolet (UV) spectroscopy revealed MgO-NPs, which could be confirmed by the narrow peak obtained at 200 nm with an absorption value of 2.5 (Fig. 6).

#### Transmission electron microscope

**Fig. 7:**
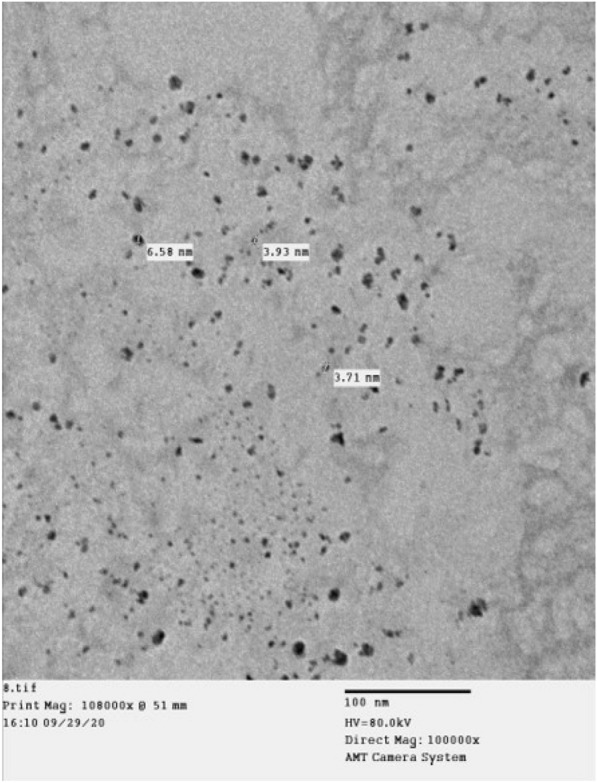
TEM photograph of MgO-NPs showing spherical shapes and particle sizes ranging from 3.71 to 6.58 nm, homogeneously distributed

The synthesized MgO-NPs had average sizes ranging from 3.71 to 6.58 nm with spherical shapes (Fig. 7).

#### Zeta potential

**Fig. 8:**
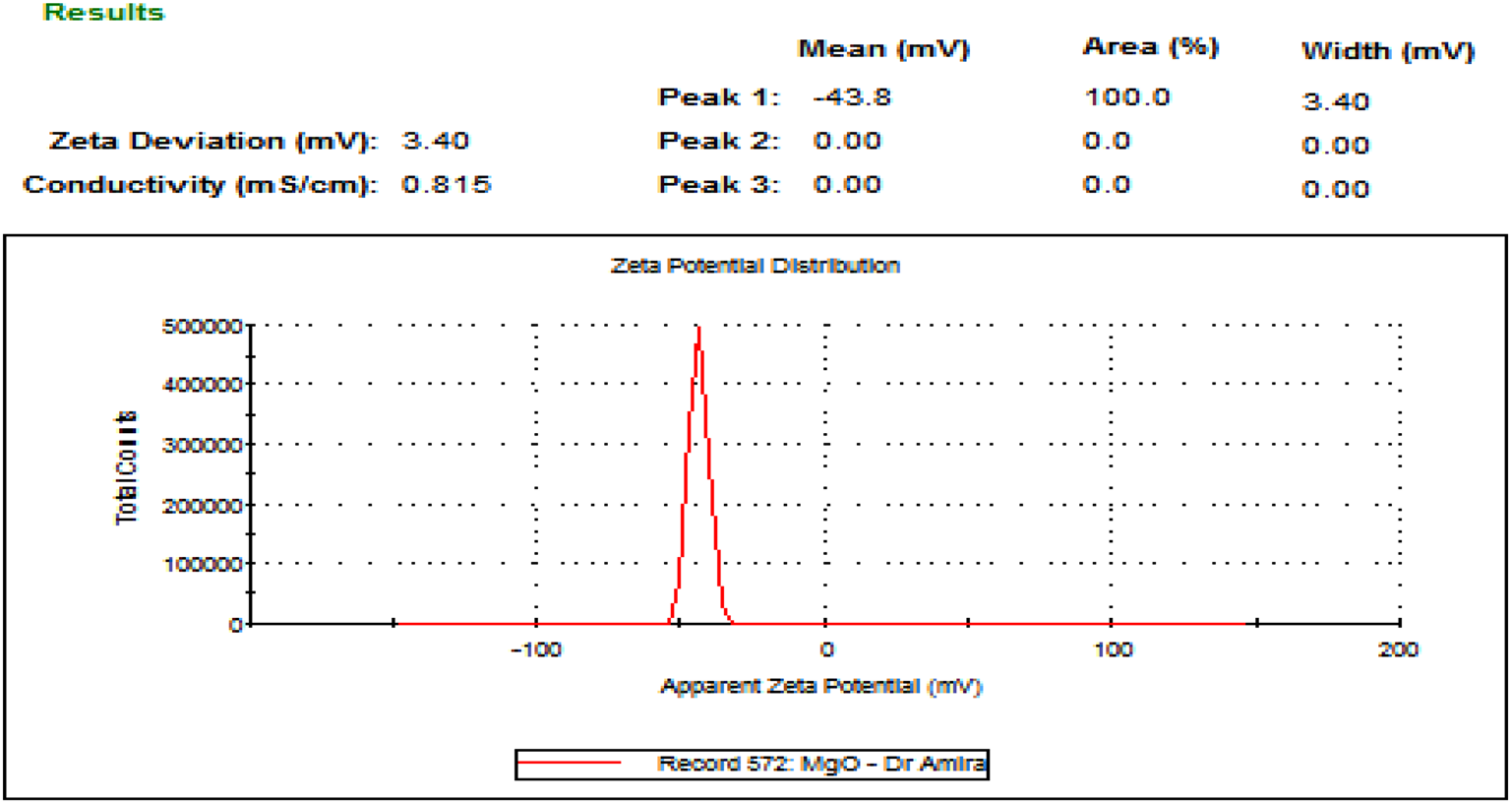
Histogram of the zeta potential distribution of MgO-NPs, showing a zeta potential of − 43 mV

The surface charge of the particles was expressed as a zeta potential of − 43.8 mV (Fig. 8), which indicated that MgO-NPs were more stable.

#### FTIR

**Fig. 9:**
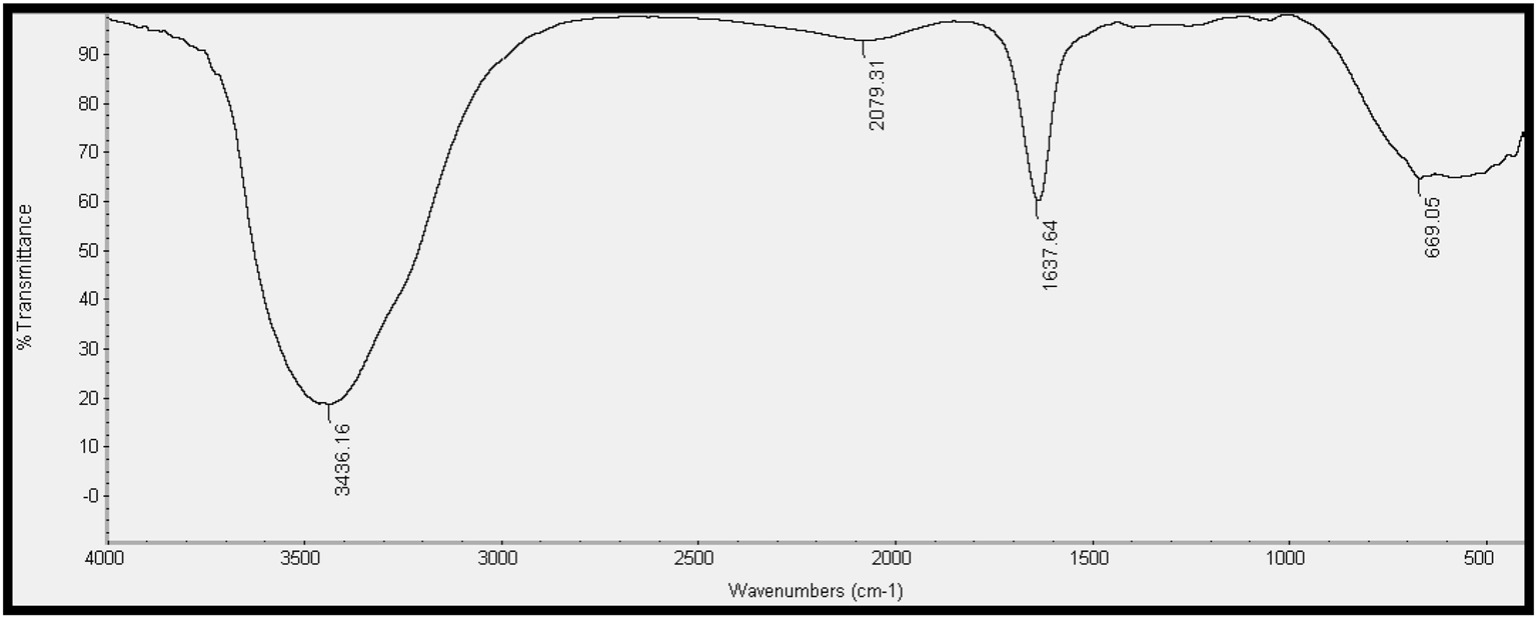
FTIR spectra of MgO-NPs measured at wavenumbers of 400–4000 cm^− 1^. Strong bands were visible at 3436.16, 2079.31, 1637.64, and 669.05 cm^− 1^

The FTIR spectrum of MgO-NPs revealed distinct absorption bands at 3436.16, 2079.31, 1637.64, and 669.05 cm^− 1^, as depicted in (Fig. 9). The band at 3436.16 cm^− 1^ is attributed to the O–H stretching of H-bonded alcohols and phenols. The band at 2079.31 cm^− 1^ is attributed to the O–H stretching of carboxylic acids. The band at 1637.64 cm^− 1^ corresponded to the NO_2_ stretching of the nitro compound and the presence of amides I and II. This band arises from the vibrations of the carbonyl stretch of the amide linkages and the N–H stretch atoms of proteins. The peak observed at 669.05 cm^− 1^ was within the fingerprint region and was complex, encompassing a multitude of distinct vibrations.

#### XRD

**Fig. 10:**
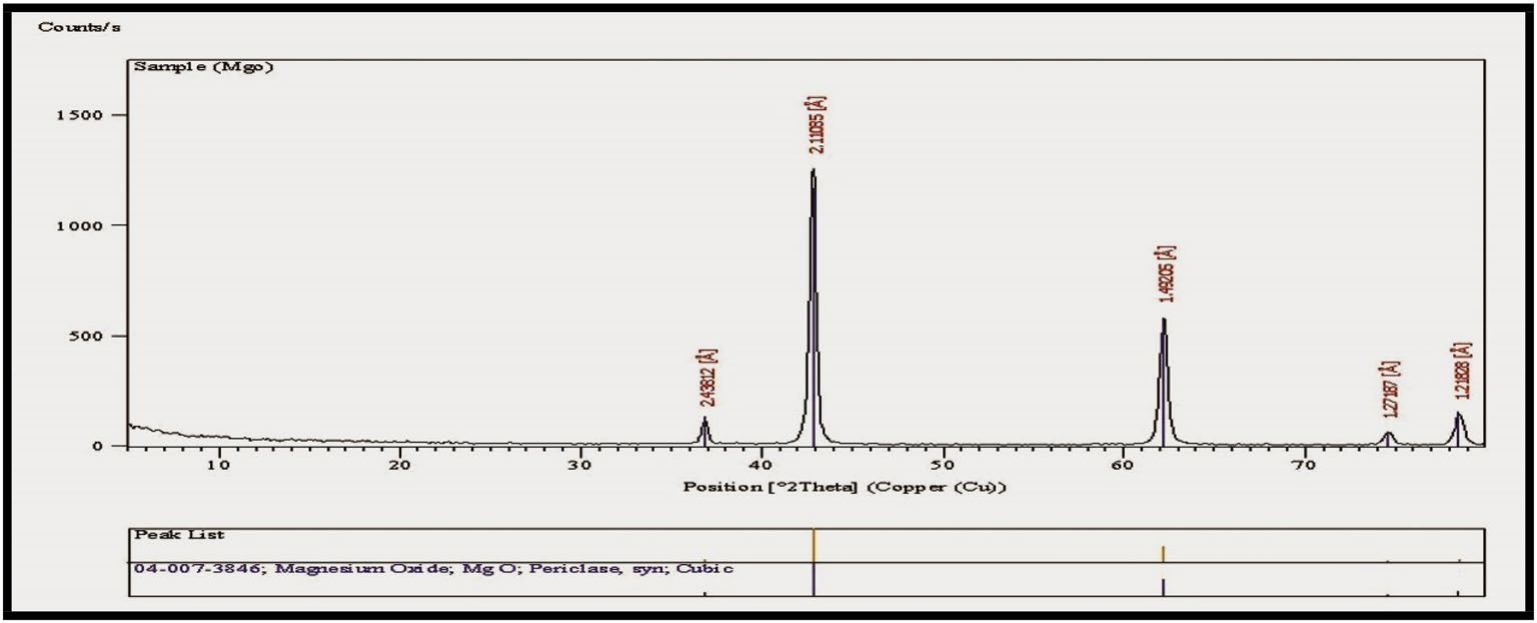
XRD spectra of the MgO nanopowder used to detect its crystalline nature. It shows two sharp peaks at 2θ of 43.006° and 62.447° with reference code (04-007-3846)

MgO is a single-phase material with a crystalline structure, as confirmed by the X-ray diffraction pattern of MgO-NPs using the reference code (04-007-3846). The diffraction pattern showed several well-defined peaks at 2θ values of 37.016°, 43.006°, 62.447°, 74.869°, and 78.822° correspond to the crystal planes (h k l) of (1 1 1), (2 0 0), (2 2 0), (3 1 1), and (2 2 2), respectively. The two sharp peaks observed were indexed to the (2 2 0) and (2 0 0) planes, as illustrated in (Fig. 10**)**.

### Effect of NPs with MICs of bulk on the growth of *R. solanacearum*

**Fig. 11:**
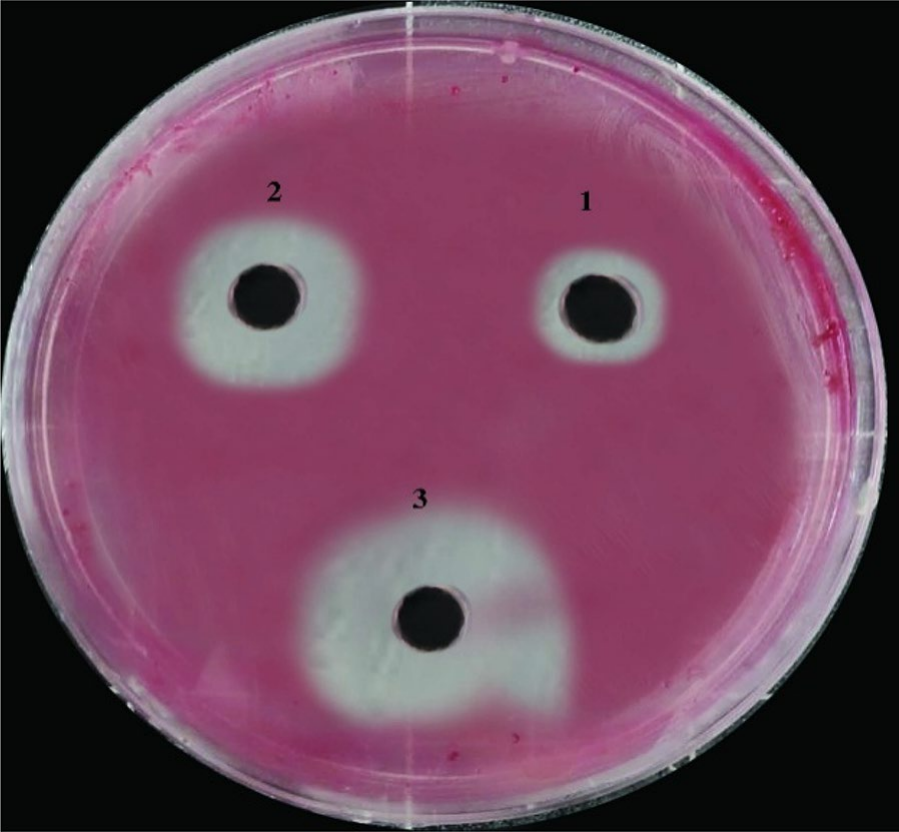
Seeded agar plate with *R. solanacearum* treated with different nanosolutions on TZC medium: (1) gentamicin, (2) MgO-NPs, and (3) CuO-NPs.

The diffusion agar technique was employed for the experimental procedure, with a well diameter of 6.0 mm and a volume of 100 µL. The antimicrobial activity of the NPs was directly proportional to the equivalent concentrations of the prepared nanosolutions. The results in (Table 2**)** and (Fig. 11**)** demonstrate that CuO-NPs exhibited the most effective inhibition of *R. solanacearum* growth, resulting in the widest zone of inhibition with an average diameter of 19.3 mm. Following CuO-NPs, MgO-NPs demonstrated significant inhibitory activity with an average zone of inhibition diameter of 17.0 mm. Notably, these nanoparticles suppressed pathogen activity considerably better than the control treatment with gentamicin.

**Table 2:** Effect of NPs with MICs of bulk against virulent and pathogenic *R. solanacearum*

Nanoparticles	Zone of Inhibition (mm)
CuO-NPs	19.3a ± 0.173
MgO-NPs	17.0b ± 0.231
Gentamicin	10.0c ± 0.288
LSD at 5%	0.23

Means ± SE, (a, b, c, ….), data with the different letter are significant, while the data with the same letter within the same column are not significantly different (p < 0.05)

Well diameter: 6.0 mm (100µL was tested)

### Synthetically produced CuO-NPs and MgO-NPs exhibited low minimum inhibitory concentrations (MICs) and minimum bactericidal concentrations (MBCs) against *R. solanacearum*

**Fig. 12:**
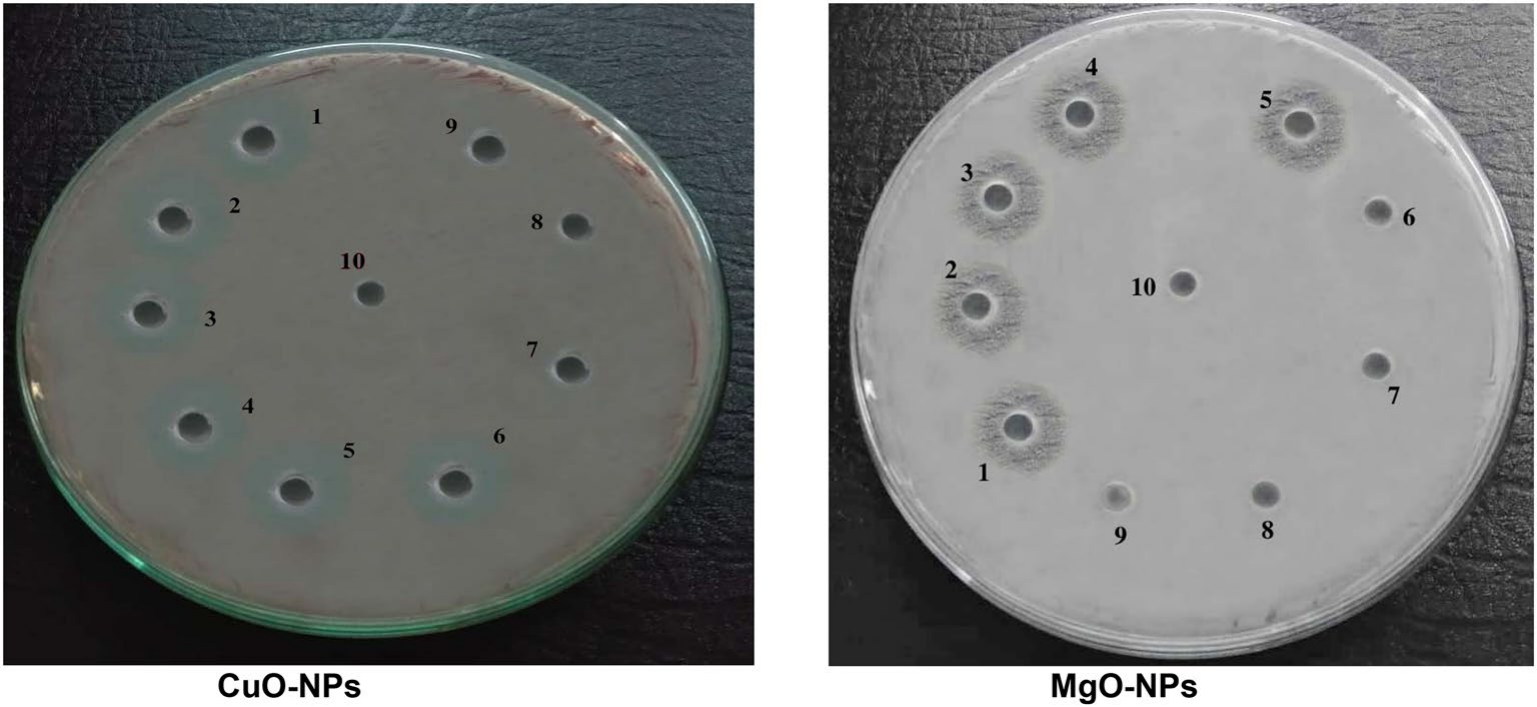
Seeded agar plates with *R. solanacearum* treated with different concentrations (mg/mL) of the prepared nanosolutions. The numbers 1–10 represent different concentrations of NPs: (1) 3 mg/mL, (2) 1.5 mg/mL, (3) 1 mg/mL, (4) 0.75 mg/mL, (5) 0.6 mg/mL, (6) 0.5 mg/mL, (7) 0.43 mg/mL, (8) 0.37 mg/mL, (9) 0.33 mg/mL and (10) 0.3 mg/mL)

The MIC and MBC of NPs are presented in (Tables 3 and 4), as well as in (Fig. 12). The MIC values were 0.5 mg/mL and 0.6 mg/mL for CuO-NPs and MgO-NPs, respectively. Furthermore, the MBC values were 0.6 mg/mL and 0.75 mg/mL for CuO-NPs and MgO-NPs, respectively.

**Table 3:** MIC and MBC of synthesized NPs against *R. solanacearum*

NPs	MIC (mg/mL)	MBC (mg/mL)
CuO-NPs	0.50b ± 0.052	0.60a ± 0.035
MgO-NPs	0.60a ± 0.046	0.75a ± 0.035
LSD at 5%	0.04	n.s

Means ± SE, (a, b,….), data with the different letter are significant, while the data with the same letter within the same column are not significantly different (p < 0.05)

**Table 4:** Antibacterial activities as growth density of different concentrations of NPs against *R. solanacearum*

Conc. (mg/mL)	Nanoparticles		
	CuO-NPs (starting with 3 mg/mL)	MgO-NPs starting with 3 mg/mL	LSD at 5%
	Log CFU/mL		
Control	9.0a ± 0.354	9.0a ± 0.424	n.s
3.00	0 ± 0.00	0 ± 0.00	0
1.50	0 ± 0.00	0 ± 0.00	0
1.00	0 ± 0.00	0 ± 0.00	0
0.75	0 ± 0.00	0 ± 0.00	0
0.60	0 ± 0.00	0 ± 0.00	0
0.50	0.0b ± 0.0	1.5a ± 0.424	0.96
0.43	1.0b ± 0.424	2.4a ± 0.283	1.15
0.38	2.43b ± 0.156	4.1a ± 0.354	0.87
0.33	3.91a ± 0.354	4.8a ± 0.495	n.s
0.30	4.33b ± 0.354	5.7a ± 0.424	1.25
0.27	5.04b ± 0.212	6.1a ± 0.283	0.80

Means ± SE, (a, b, …), data with the different letter are significant, while the data with the same letter within the same column are not significantly different (p < 0.05)

### Effect of NPs and bulks at MICs on total lipids, phospholipids, and neutral lipids of *R. solanacearum*

Table 5 shows a decrease in total lipid and neutral lipid levels in *R. solanacearum*, accompanied by an increase in phospholipid levels under all treatment conditions. Specifically, total lipid and neutral lipid levels were measured at 1.93% and 37.1% in the CuO-NPs treatment and at 2.03% and 39.7%, respectively, in the MgO-NPs treatment. In contrast, phospholipid levels increased to 62.9% and 60.3% in the CuO-NPs and MgO-NPs treatments, respectively, compared to 41.8% in untreated cells.

**Table 5:** Effect of NPs and bulks on MICs of total lipids, phospholipids, and neutral lipids in *R solanacearum*

Lipids %	Treatments at MIC conc.						
	Control	Gentamicin	CuSO_4_	MgSO_4_	CuO-NPs	MgO-NPs	LSD at 5%
Total lipids (TL) as % of dry weight	4.60a ± 0.057	2.51d ± 0.053	3.01c ± 0.045	3.18b ± 0.041	1.93e ± 0.037	2.03e ± 0.024	0.16
Phospholipids (as % of TL)	41.8f ± 0.057	54.40c ± 0.086	50.16d ± 0.029	47.60e ± 0.057	62.90a ± 0.033	60.30b ± 0.061	0.18
Neutral lipids (as % of TL)	58.20a ± 0.057	45.60d ± 0.065	49.840c ± 0.053	52.40b ± 0.057	37.10f ± 0.053	39.70e ± 0.061	0.20

Means ± se, (a, b, c, d, ….), data with the different letter are significant, while the data with the same letter within the same row are not significantly different (p < 0.05)

### Effect of NPs on sub-cellular structures

**Fig. 13:**
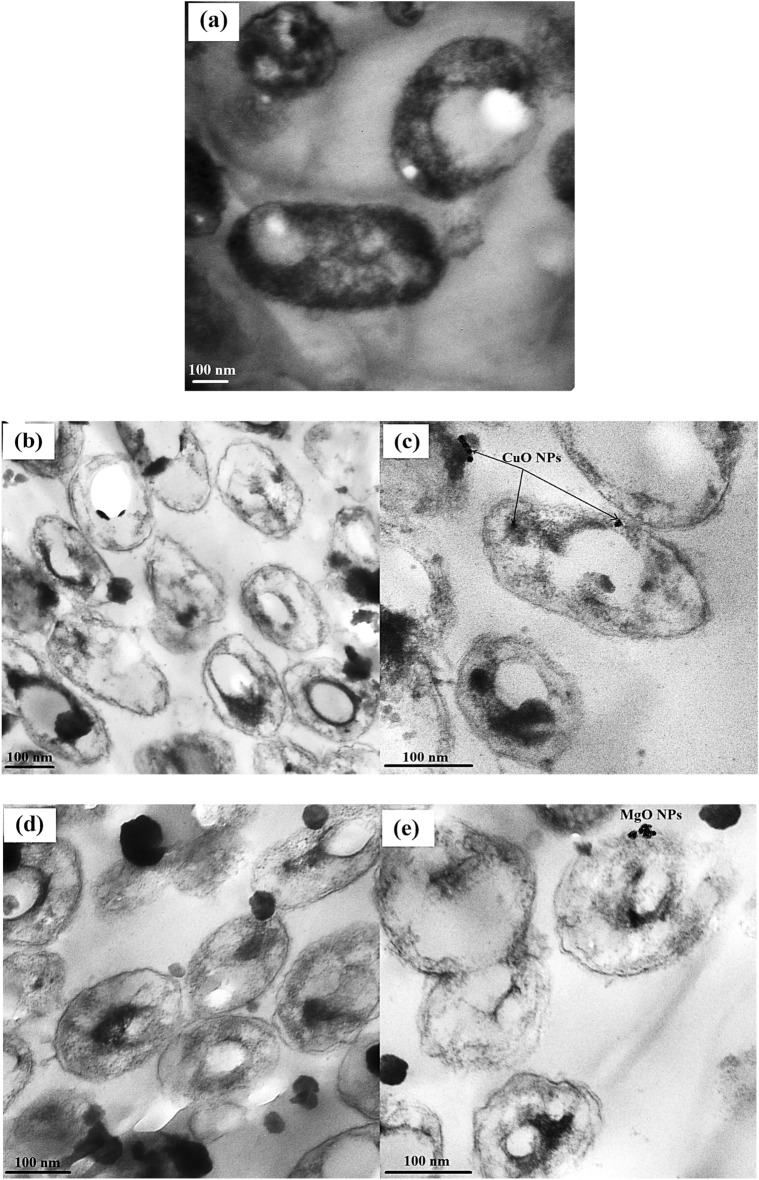
Photographs showing the mode of action of NPs against *R. solanacearum*: **a** control cells without NPs, **b** cells treated with CuO-NPs, **c** the magnified cells treated with CuO-NPs, **d** cells treated with MgO-NPs, **e** the magnified cells treated with MgO-NPs

TEM visualization revealed the ultrastructures of both untreated and treated bacterial cells, providing an opportunity to observe the morphological changes between treated and untreated *R. solanacearum* (Fig. 13). Cross-sectional photographs of untreated cells demonstrated intact membrane integrity after 24 h. In contrast, cells treated with NPs exhibited random and tight NP adherence to the cell surface, visibly disrupting the cell wall. Furthermore, NPs stimulated the formation of multiple vesicles emanating from the cell wall, resulting in significant damage and cell lysis. The treated cells showed substantial disruption accompanied by major structural changes and outer membrane damage. The NPs induced irregular gaps in the outer membrane, increasing permeability and releasing lipopolysaccharides. Additionally, large portions of NPs became trapped within the cell walls and accumulated inside the cells, causing significant damage to vital components.

### Effect of NPs mixed in compost tea or humic acid on the inhibition of *R. solanacearum *using seed dressing with tubers soaked in *R. solanacearum*

Bacterial enumeration was performed on cultivated soil samples infected with *R. solanacearum* and infected soil samples treated with compost tea and humic acid supplemented with NPs. Enumeration was conducted using TZC agar medium, and the viable counts of the isolated bacteria are presented in (Table 6. The results indicated that samples treated with humic acid exhibited lower levels of microorganisms than those treated with compost tea. The control group had the highest bacterial count (CFU/g dry wt).

**Table 6:** Total viable counts of *R. solanacearum* in infected soil samples

Treatment	Log CFU/g. Soil	
	CuO-NPs	MgO-NPs
Compost	3.83b ± 0.002	4.30b ± 0.007
Humic acid	2.80b ± 0.005	3.00c ± 0.003
Control	7.01a ± 0.005	7.01a ± 0.007
LSD at 5%	1.05	0.008

Means ± se, (a, b, c, d, ….), data with the different letter are significant, while the data with the same letter within the same row are not significantly different (p < 0.05)

### Influence of NPs and bulk materials at their respective MICs as seed dressing with tubers on potato brown rot incidence and tuber yields

**Fig. 14:**
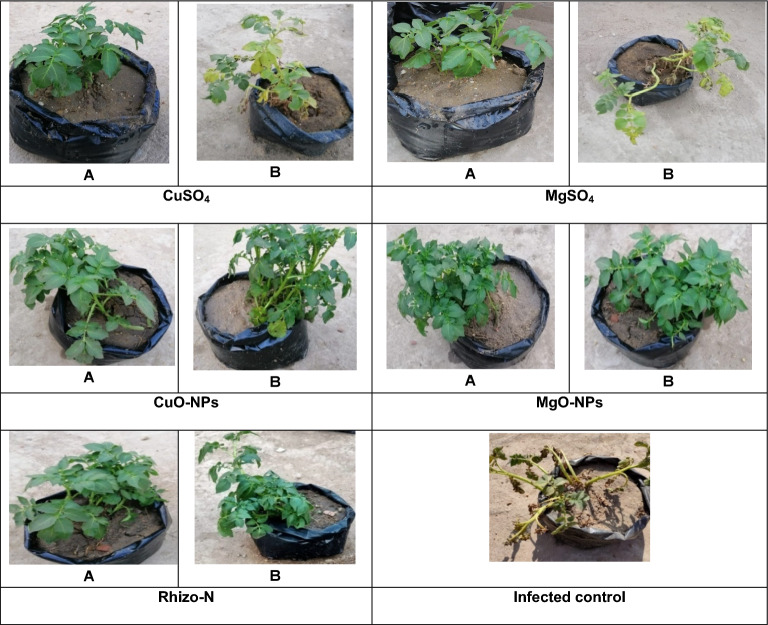
**A** Healthy plants without intervention and **B** treated plants. Bacterial wilt disease symptoms in the infected control plants were particularly severe. In contrast, the disease indices of potato plants exposed to CuO-NPs or MgO-NPs showed a decrease in disease incidence compared to bulk CuSO_4_ and bulk MgSO_4_

The data presented in (Table 7**)** and illustrated in (Fig. 14**)** demonstrate the effectiveness of metal oxide NPs, specifically CuO-NPs, and MgO-NPs, in reducing disease incidence compared to the control treatment. CuO-NPs and MgO-NPs demonstrated disease reductions of 71.2% and 69.4%, respectively. In contrast, the application of magnesium sulfate at a concentration of 3 mg/mL resulted in the lowest disease reduction (39.5%) compared to the control treatment.

**Table 7:** Influence of NPs and bulks used as seed dressing with tubers at their respective MICs on potato brown rot incidence and tuber yields

Bulks & NPs	Treatment	Tuber number	Tuber weight	Disease index (DI) %	Disease reduction (Efficacy) %
CuSO _4_	Healthy	11.30e ± 0.061	140.70b ± 0.200	0.00 g ± 0.000	100.00a ± 0.00
	Treated	6.60 h ± 0.061	70.00 g ± 0.253	57.00c ± 0.094	43.00e ± 0.229
MgSO _4_	Healthy	13.70a ± 0.057	142.30a ± 0.220	0.00 g ± 0.000	100.00a ± 0.00
	Treated	6.00i ± 0.053	76.00f ± 0.237	60.50b ± 0.098	39.50f ± 0.245
CuO-NPs	Healthy	12.50c ± 0.037	142.30a ± 0.184	0.00 g ± 0.000	100.00a ± 0.00
	Treated	10.30f ± 0.049	141.70a ± 0.237	28.80f ± 0.065	71.20b ± 0.261
MgO-NPs	Healthy	13.00b ± 0.037	138.00c ± 0.310	0.00 g ± 0.000	100.00a ± 0.00
	Treated	11.80d ± 0.037	136.30d ± 0.265	30.60e ± 0.106	69.40c ± 0.163
*Rhizo-N		9.00 g ± 0.049	103.00e ± 0.216	44.40d ± 0.102	55.60d ± 0.204
Positive control (infected)		4.00j ± 0.024	60.00 h ± 0.384	100.00a ± 0.000	00.0 g ± 0.00
LSD at 5%		0.12	0.88	0.21	0.63

Means ± SE, (a, b, c, d, ….), data with the different letter are significant, while the data with the same letter within the same column are not significantly different (p < 0.05)

*Rhizo-N: commercial biocide; Biotech for fertilizers and biocides, Egypt

### Influence of NPs and bulks at their respective MICs and used as seed dressing with tubers on pigment contents of potato plants infected with *R. solanacearum*

The observed chlorophyll (a) values were 0.369 and 0.442 when CuSO_4_ and MgSO_4_ were added to the soil, respectively. The values of chlorophyll (b) were 0.221 and 0.351 in the CuSO_4_ and MgSO_4_ treatments, respectively. Among the treatments, the lowest carotenoid values were recorded at 0.120 in the presence of CuSO_4_. In contrast, when CuO-NPs and MgO-NPs were introduced into the soil, the highest values of chlorophyll (a) were 0.769 and 0.642, respectively, whereas the chlorophyll (b) values reached 0.621 and 0.551, respectively. These values were notably higher than those observed for the control treatments **(**Table 8**)**.

**Table 8:** Influence of NPs and bulks at their respective MICs used as seed dressing with tubers on pigment content (mg/g of fresh leaf weight) of potato plants infected with *R. solanacearum*

Bulks & NPs	Treatment	Chlorophyll (a)	Chlorophyll (b)	Carotenoids
		mg\g (fwt)		
CuSO _4_	Healthy	0.476 g ± 0.002	0.301 h ± 0.003	0.200 g ± 0.002
	Treated	0.369i ± 0.003	0.221i ± 0.002	0.120 h ± 0.002
MgSO _4_	Healthy	0.529f ± 0.003	0.401f ± 0.003	0.496c ± 0.002
	Treated	0.442 h ± 0.002	0.351 g ± 0.002	0.424d ± 0.002
CuO-NPs	Healthy	0.876a ± 0.002	0.691a ± 0.002	0.411e ± 0.002
	Treated	0.769c ± 0.003	0.621b ± 0.002	0.392f ± 0.003
MgO-NPs	Healthy	0.799b ± 0.002	0.601c ± 0.003	0.552b ± 0.002
	Treated	0.642d ± 0.002	0.551d ± 0.002	0.654a ± 0.002
*Rhizo-N		0.596e ± 0.002	0.504e ± 0.002	0.420d ± 0.002
Positive control (infected)		0.218j ± 0.003	0.191j ± 0.002	0.093i ± 0.002
LSD at 5%		0.010	0.010	0.008

Means ± SE, (a, b, c, d, ….), data with the different letter are significant, while the data with the same letter within the same column are not significantly different (p < 0.05)

*Rhizo-N: commercial biocide; Biotech for fertilizers and biocides, Egypt

### Effect of NPs and bulk treatments at their respective MICs used as seed dressing with tuber on oxidative enzyme activities in potato plants

Table 9 presents the peroxidase activity values obtained under different treatments. The highest peroxidase activity values of 0.624 and 0.606 were recorded by adding CuO-NPs and MgO-NPs, respectively. These values were nearly within the range observed for healthy plants. In contrast, the lowest peroxidase activity values were observed when bulk MgSO_4_ and CuSO_4_ were added (0.176 and 0.351, respectively). These values were significantly lower than that of the Rhizo-N treatment (0.401).

Furthermore, the maximum polyphenol oxidase activity values were observed by adding CuO-NPs and MgO-NPs, measuring 0.405 and 0.427, respectively. These values were also within the range typically observed in healthy plants. In contrast, the lowest polyphenol oxidase activity values were recorded when bulk CuSO_4_ and MgSO_4_ were added (0.129 and 0.138, respectively). These values were notably lower than that of the the Rhizo-N treatment (0.274).

**Table 9:** Effects of NPs and bulk treatments at their respective MICs and used as seed dressing with tubers on the activities of oxidative enzymes in potato plants

Bulks & NPs	Treatment	Enzyme activity as OD/mg/g fresh weight/min	
		Peroxidase activity (PO)	Polyphenol oxidase activity (PPO)
CuSO _4_	Healthy	0.363e ± 0.003	0.145 g ± 0.002
	Treated	0.351e ± 0.004	0.129i ± 0.002
MgSO _4_	Healthy	0.194f ± 0.004	0.178f ± 0.003
	Treated	0.176 g ± 0.002	0.138gh ± 0.003
CuO-NPs	Healthy	0.653a ± 0.003	0.418c ± 0.002
	Treated	0.624b ± 0.002	0.405d ± 0.002
MgO-NPs	Healthy	0.644a ± 0.003	0.462a ± 0.002
	Treated	0.606c ± 0.004	0.427b ± 0.002
*Rhizo- N		0.401d ± 0.005	0.274e ± 0.002
Positive control (infected)		0.164 g ± 0.005	0.133hi ± 0.002
LSD at 5%		0.014	0.007

Means ± se, (a, b, c, d,….), data with the different letter are significant, while the data with the same letter within the same column are not significantly different (p < 0.05)

*Rhizo-N: commercial biocide; Biotech for fertilizers and biocides, Egypt

### Effect of NPs and bulk treatments at their respective MICs used as seed dressing with tubers on phenol activities in potato plants

The phenol contents were examined and are presented in Table 10, demonstrating an increase in all tested treatments compared with the untreated control. The highest phenolic content was observed when adding CuO-NPs and MgO-NPs, measuring 4.368 and 3.091, respectively. These values were within the range typically observed in healthy plants. In contrast, the lowest phenolic content values were recorded when bulk MgSO_4_ and CuSO_4_ were added (2.183 and 2.325, respectively). These values were notably lower than that of the Rhizo-N treatment (2.208).

**Table 10:** Effects of NPs and bulk treatments at their respective MICs used as chemical inducers on phenol activity in potato plants

Bulks & NPs	Total phenol (mg/g fwt)	
	Healthy	Treated
CuSO _4_	2.600e ± 0.009	2.325f ± 0.004
MgSO _4_	2.003 h ± 0.004	2.183 g ± 0.004
CuO-NPs	4.204b ± 0.004	4.368a ± 0.006
MgO-NPs	3.003d ± 0.004	3.091c ± 0.004
*Rhizo- N	2.208 g ± 0.005	
Positive control (infected)	0.441i ± 0.006	
LSD at 5%	0.034	

Means ± se, (a, b, c, d,….), data with the different letter are significant, while the data with the same letter within the same column are not significantly different (p < 0.05)

*Rhizo-N: commercial biocide; Biotech for fertilizers and biocides, Egypt

## Discussion

The bacterium *R. solanacearum*, as identified by Yabuuchi et al. (1995), is responsible for potato brown rot, and its presence in Egypt has long been documented (Briton-Jones 1926). This disease poses considerable quarantine challenges when Egyptian table potatoes are exported to Europe (Tomlinson et al. 2009). In this study, chemically produced CuO and MgO metal oxide nanoparticles effectively suppressed potato brown rot disease.

The results obtained in this study indicated a complete reduction (100%) in bacterial growth, as determined by the number of colony-forming units (CFU), with an increase in metal concentration. In contrast, lower metal concentrations did not cause any reduction in CFU numbers compared with the control treatment. These findings are consistent with those reported by Cai et al. (2018), who observed that bulk MgO doses exhibited high minimum inhibitory and MBCs values.

The application of ultraviolet (UV) visible spectroscopy in this study revealed that CuO-NPs exhibited a surface plasmon absorption band with a peak at 220 nm and an absorption value of approximately 0.2. These results align closely with the findings of Fadhil et al. (2018), who reported that the UV-visible spectra of CuO-NPs typically fall within the wavelength range of 200–300 nm. TEM analysis of the CuO-NPs demonstrated that the particles were very small, with sizes of 3.59 and 6.05 nm. They appeared smooth, spherical, loosely distributed, and uniformly distributed within the matrix. These observations are consistent with those of Khashan et al. (2016), who also observed CuO-NPs as spherical particles ranging from 3.0 to 40.0 nm.

Furthermore, CuO-NPs possessed a higher surface charge, as indicated by a zeta potential value of -39.5 mV. This higher stability of CuO-NPs was supported by Song et al. (2016), who reported zeta potential values of -14.9 and − 18 mV in culture media. Additionally, FTIR spectroscopy analysis of the CuO-NPs in this study showed strong absorption bands at 3440.7, 1636.78, and 432.11 cm^− 1^, suggesting the presence of different chemical compounds surrounding the metal nanoparticles. These findings align well with those reported by Pandiyarajan et al. (2013), who confirmed that copper NPs exhibited three distinct bands corresponding to Cu-O bond vibrations.

The XRD pattern of the synthesized CuO-NPs confirmed their identity, exhibiting characteristic peaks at h k l (0 0 2), (1 1 –1), (1 1 1), (2 0 0), and (2 0 –2). These results closely correspond to the findings reported by Mobarak et al. (2022), providing further support for the characterization of CuO-NPs in this study.

Ultraviolet-visible (UV-vis) spectroscopy of the MgO-NPs revealed a surface plasmon resonance (SPR) absorption band within the 200–300 nm peak range, with an absorption value of approximately 0.6. Essien et al. (2019) identified the maximum SPR for MgO-NPs at 260 nm. High-resolution transmission electron microscopy (HR-TEM) images revealed the particulate nature of MgO-NPs, exhibiting small sizes (3.71 and 6.58 nm) and a smooth, spherical morphology. These particles were loosely and uniformly distributed throughout the matrix. Similarly, Cai et al. (2018) indicated that MgO-NPs were spherical, with particle sizes ranging from 5 to 17 nm. The zeta potential of − 43.8 mV observed in this study suggested higher stability for MgO-NPs. Cai et al. (2018) emphasized the significant impact of zeta potential on the antibacterial activity of NPs, highlighting its influence on particle size. In the case of the MgO-NPs, FTIR spectroscopy revealed strong absorption bands at 3436, 2079.31, 1637.64, and 669.05 cm^− 1^. The peak at 669.05 cm^− 1^ represents a complex fingerprint region encompassing multiple distinct vibrations. The FTIR spectrum of the magnesium nanoparticles indicated the presence of various chemical compounds surrounding them. This finding aligns with a previous conclusion drawn by Imani and Safaei (2019) that several organic compounds contain magnesium nanoparticles.

The diffraction pattern obtained from the X-ray analysis of the prepared MgO-NPs in the present study demonstrated that MgO had a single-phase crystalline structure indexed to the (1 1 1), (2 0 0), (2 2 0), (3 1 1), and (2 2 2). The two sharp peaks observed were indexed to the (2 2 0) and (2 0 0) diffraction planes. Previous studies conducted by Imani and Safaei (2019) and Maji et al. (2020) documented distinctive reflection peaks in the diffraction pattern of as-prepared MgO-NPs, indexed to the (1 1 1), (2 0 0), (2 2 0), (3 1 1), and (2 2 2) diffraction planes.

The antibacterial activities of the CuO-NPs and MgO-NPs synthesized in this study were investigated. The results demonstrated that CuO-NPs exhibited the highest antibacterial activity, with an inhibitory zone of 19.3 mm on TZC media, surpassing gentamicin. Pandiyarajan et al. (2013) reported that *Shigella flexneri* and *B. subtilis*, depicted high sensitivity indicated by the inhibitory zones created by CuO-NPs. Similarly, Cai et al. (2017) found that MgO-NPs exhibited broad-spectrum direct toxicity against various pathogens, including gram-positive (*S. aureus*) and gram-negative (*E. coli*) bacteria.

Furthermore, El-Batal et al. (2020) documented that CuO-NPs combined with streptomycin demonstrated substantial inhibition of biofilm formation, reaching approximately 90.99%, 84.23%, and 83.42% against *Clavibacter michiganensis* subsp. *sepedonicus*, *R. solanacearum*, and *Dickeya solani*, respectively. The present data indicate that the prepared nanoparticles exhibited stronger antibacterial activity than bulk materials at the same concentration, particularly against *R. solanacearum*. These findings are consistent with the observations of Mondal and Mani (2012), who reported that nanoparticles showed superior antibacterial activity compared to bulk materials when tested at equivalent concentrations.

In the present study, MIC and MBC were determined using the microdilution method. The results revealed that the MIC value of CuO-NPs against *R. solanacearum* was 0.5 mg/mL, whereas the MIC value of MgO-NPs was slightly higher at 0.6 mg/mL. This indicated that the CuO-NPs exhibited higher antibacterial activity, as nearly all *R. solanacearum* cells were eradicated at this concentration. Moreover, several researchers confirmed the MICs of CuO-NPs.

Mahapatra et al. (2008) demonstrated the susceptibility of *Shigella*, *Salmonella paratyphi*, *Pseudomonas aeruginosa*, and Klebsiella pneumoniae bacteria to CuO nanoparticle dispersion with sizes ranging from 80 to 100 nm. Chen et al. (2019) illustrated that exposure to 250 mg/mL of CuO-NPs resulted in the death of all *R. solanacearum* cells. Furthermore, Cai et al. (2018) reported that *R. solanacearum* growth was suppressed by MgO-NPs at concentrations ranging from 50 to 250 mg/mL, with MIC and MBC values of 200 and 250 mg/mL, respectively.

In this study, lipid analysis was conducted to investigate the effect of cell material leakage on respiratory function and subsequent cell death. The results demonstrated that total lipids and neutral lipids in *R. solanacearum* decreased, whereas phospholipids increased in all treatments at MICs. The CuO-NPs exhibited the highest efficacy in reducing total and neutral lipids. These findings align with those of Azam et al. (2012), Hemeg (2017), and Xin et al. (2019), who suggested that ions generated by nanoparticles could lead to protein denaturation by interacting with and disrupting the negatively charged bacterial cell wall, ultimately resulting in cell death and impaired respiratory activity. Previous studies have highlighted the utilization of inorganic metal NPs, such as ZnO, Ag, TiO_2_, and Cu, as antibacterial agents, attributed to the generation of reactive oxygen species (ROS), which can damage various biological components, including proteins, lipids, and nucleic acids (Hemeg 2017).

Using TEM, we investigated the morphological structure of *R. solanacearum* treated with NPs. These findings revealed substantial alterations in the bacterial cells, with notable changes and damage to the outer membrane. The NPs induced irregular gaps in the outer membrane, enhancing permeability and leading to the release of LPS. Furthermore, a significant amount of NPs was trapped within the cell walls and accumulated inside the cells, resulting in critical damage. These results are consistent with those of previous studies that examined the intricate mechanisms through which metal NPs exert harmful effects on bacteria and elucidated the interaction between bacteria and nanoparticles. These processes could be attributed to the large surface area of adhesins, which encompasses diverse bacterial surface glycoprotein receptors (Jeevanandam and Klabunde 2002), as well as the electrostatic interactions between negatively charged cell membranes and positively charged nanoparticles, as explained by Raffi et al. (2010). Additionally, the antibacterial effect could be attributed to DNA damage induced by ROS, which significantly impairs cell viability (Chen et al. 2019).

Moreover, the application of NPs to humic acid and compost tea influenced the viable counts of bacteria isolated from infected soil samples. Specifically, the highest bacterial counts were observed in the compost tea samples compared to the humic acid samples and the control group (CFU/g dry wt).

The greenhouse experiment involved the application of NPs, which resulted in a significant reduction of bacterial wilt. Specifically, CuO-NPs and MgO-NPs reduced bacterial wilt by 71.2% and 69.4%, respectively. In the absence of nanoparticle treatment, potato tubers exhibited nearly complete mortality. These findings are consistent with those reported by Chen et al. (2019), who observed that exposure to CuO-NPs at doses of 50, 125, and 250 mg/mL reduced the disease indices of tobacco seedlings by 74.2%, 62.2%, and 38.1%, respectively, demonstrating the development of systemic resistance and various disease symptoms.

Khan and Siddiqui (2018) investigated the use of ZnO-NPs at two different concentrations, applied through spraying, to manage the eggplant disease complex caused by *Phomopsis vexans*, *R. solanacearum*, and *Meloidogyne incognita*. In addition, researchers have employed seed and soil inoculation methods. The application of 200 ppm ZnO-NPs by spraying resulted in a significant decrease in the disease index, reducing it to one.

Consequently, the utilization of various NPs, as demonstrated in the aforementioned studies, has shown promising potential for increasing crop yields compared to lower concentrations of equivalent bulk materials.

In this study, applying CuO-NPs and MgO-NPs to the soil resulted in the highest recorded values of chlorophyll (a) and chlorophyll (b), respectively, compared with the lower values observed with equivalent bulk materials. The use of NPs significantly increased the chlorophyll content, indicating improved plant photosynthetic activity. Previous studies have indicated that nanoparticles can enhance root pore formation, nutrient uptake, and hydromineral flow (Castiglione et al. 2011). Furthermore, Singh et al. (2021) reported that NPs contributed to increased nutrient absorption and chlorophyll content in plants.

Moreover, the present data revealed that the application of NPs led to increased peroxidase and polyphenol oxidase activities, as well as enhanced phenolic content. The highest peroxidase and polyphenol oxidase activities were recorded when CuO-NPs and MgO-NPs were used, respectively, and these values were comparable to those observed in healthy plants. Conversely, adding CuSO_4_ and MgSO_4_ bulk materials resulted in the lowest activity values for these enzymes.

## Conclusions

This study was designed to reduce the reliance on pesticides. It focused on comparing and discussing the antibacterial effects of CuO and MgO NPs against *R. solanacearum*, a phytopathogenic bacterium that causes destructive bacterial wilt. A straightforward chemical process was employed to synthesize CuO-NPs and MgO-NPs. The NPs produced were characterized using UV and zeta potential measurements, TEM, and FTIR spectroscopy. Additionally, a parallel experiment was conducted to examine the impact of copper and magnesium sulfate solutions. The experimental data revealed that both CuO-NPs and MgO-NPs exhibited significantly stronger antibacterial activity, with CuO-NPs demonstrating greater biocompatibility. CuO-NPs exhibited lower MIC and MBC values than MgO-NPs.

Furthermore, TEM imaging revealed that the primary toxicity mechanism involved a rupture of the outer cell membrane, resulting in cellular protein damage. In vivo, the application of CuO-NPs and MgO-NPs influenced the growth of potato plants by reducing the disease index. CuO-NPs and MgO-NPs exhibited control efficacy of 71.2% and 69.4%, respectively. Given their low dosage requirements and potent antibacterial activity, both CuO-NPs and MgO-NPs hold promise as potential biocides for effective plant disease control and offer alternative agricultural disease management strategies. Future research should focus on understanding nanoparticle absorption and transport within the rhizosphere.

## Data Availability

The article includes all the necessary information and presents the original contributions discussed in this study. For more information, please get in touch with the corresponding authors.
